# Oxidative cyclization of *N*-methyl-dopa by a fungal flavoenzyme of the amine oxidase family

**DOI:** 10.1074/jbc.RA118.004227

**Published:** 2018-09-07

**Authors:** Majd Lahham, Tea Pavkov-Keller, Michael Fuchs, Johannes Niederhauser, Gabriel Chalhoub, Bastian Daniel, Wolfgang Kroutil, Karl Gruber, Peter Macheroux

**Affiliations:** From the ‡ Institutes of Biochemistry, Graz University of Technology, Petersgasse 12/II, 8010 Graz,; the § Institute of Molecular Biosciences, University of Graz, Humboldtstrasse 50, 8010 Graz, and; the ¶ Institute of Chemistry, University of Graz, Heinrichstrasse 28/2, 8010 Graz, Austria

**Keywords:** oxidation-reduction (redox), flavoprotein, flavin adenine dinucleotide (FAD), X-ray crystallography, biosynthesis, amine oxidase, fructose amine oxidase, fumisoquin biosynthesis, isoquinolines, oxidative cyclization

## Abstract

Flavin-dependent enzymes catalyze many oxidations, including formation of ring structures in natural products. The gene cluster for biosynthesis of fumisoquins, secondary metabolites structurally related to isoquinolines, in the filamentous fungus *Aspergillus fumigatus* harbors a gene that encodes a flavoprotein of the amine oxidase family, termed *fsqB* (fumisoquin biosynthesis gene B). This enzyme catalyzes an oxidative ring closure reaction that leads to the formation of isoquinoline products. This reaction is reminiscent of the oxidative cyclization reported for berberine bridge enzyme and tetrahydrocannabinol synthase. Despite these similarities, amine oxidases and berberine bridge enzyme–like enzymes possess distinct structural properties, prompting us to investigate the structure–function relationships of FsqB. Here, we report the recombinant production and purification of FsqB, elucidation of its crystal structure, and kinetic analysis employing five putative substrates. The crystal structure at 2.6 Å resolution revealed that FsqB is a member of the amine oxidase family with a covalently bound FAD cofactor. *N*-methyl-dopa was the best substrate for FsqB and was completely converted to the cyclic isoquinoline product. The absence of the *meta*-hydroxyl group, as *e.g.* in l-*N*-methyl-tyrosine, resulted in a 25-fold lower rate of reduction and the formation of the demethylated product l-tyrosine, instead of a cyclic product. Surprisingly, FsqB did not accept the d-stereoisomer of *N*-methyltyrosine, in contrast to *N*-methyl-dopa, for which both stereoisomers were oxidized with similar rates. On the basis of the crystal structure and docking calculations, we postulate a substrate-dependent population of distinct binding modes that rationalizes stereospecific oxidation in the FsqB active site.

## Introduction

Flavin-dependent enzymes catalyze a broad range of oxidations including reactions that entail the formation of rings in natural product biosynthesis, such as tetrahydrocannabinol and isoquinoline alkaloids (reviewed in Ref. 1). Thus far, oxidative cyclization reactions appeared to be confined to flavoenzymes belonging to a protein family featuring a distinct FAD binding domain (FAD_binding_4; PF01565), such as tetrahydrocannabinol synthase (THCS)^2^ and berberine bridge enzyme (BBE) (2, 3). Recently, it was found that a biosynthetic gene cluster of *Aspergillus fumigatus* harbors a gene, termed *fsqB* (fumisoquin biosynthesis gene B) that encodes a flavoprotein belonging to the family of amine oxidases and catalyzes an oxidative cyclization yielding an isoquinoline ring system via C–C bond formation (4). Flavin-dependent amine oxidases have a well defined structural topology that diverges substantially from the BBE-like enzyme family. On the other hand, covalent linkage of the FAD cofactor to the protein backbone was reported for both the amine oxidases (*e.g.* monomeric sarcosine oxidase (MSOX) and *N*-methyl-tryptophan oxidase (MTOX)) and the family of BBE-like enzymes (*e.g.* BBE and THCS). Typically, the oxidation of *N*-methyl groups by members of the flavin-dependent amine oxidases results in the formation of an imine, which subsequently hydrolyzes to the free amino group and formaldehyde, which is often trapped by tetrahydrofolate, for example in dimethylglycine dehydrogenase. Thus, the reaction reported for FsqB diverges from the canonical reaction scheme because the imine is not hydrolyzed but subject to nucleophilic attack by the catechol moiety of the substrate. In this sense, the reaction catalyzed by FsqB may be similar to those performed by BBE and THCS (4). This analogy in the outcome of the reactions, *i.e.* oxidative cyclization of the substrate, prompted us to investigate whether FsqB also shares the mechanism of action with the enzymes of the BBE-like enzyme family. Thus, we initiated the biochemical and structural characterization of the enzyme to pave the way for a more detailed understanding of FsqB. Here, we report the crystal structure of FsqB and kinetic parameters of the WT enzyme using a set of five putative substrate analogs. Based on the 3D structure of the enzyme and docking simulations using the natural substrate, as well as substrate analogs, we have generated protein variants to further analyze the role of specific active site amino acid residues. Our study showed that FsqB structurally belongs to the amine oxidase family of flavoproteins and has an active site that is similar to well characterized enzymes of this family, notably MSOX, MTOX, and NikD. Furthermore, we could identify important amino acid residues in the active site of FsqB that participate in the reductive and oxidative half-reaction, as well as in substrate binding. Thus, FsqB is the first member of the amine oxidase family, which carries out an oxidative cyclization reaction, and we anticipate that many more members of this family will be discovered in natural product biosynthesis in the future.

## Results

### Production, purification, and characterization of FsqB

**Figure 1.**
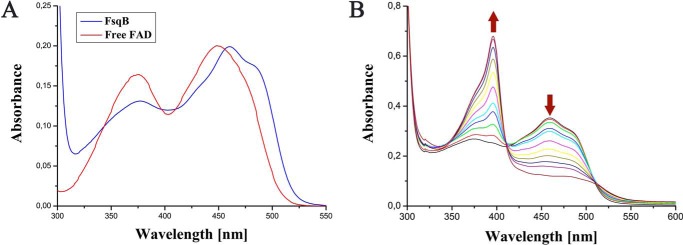
*A*, UV-visible absorption spectrum of FsqB (*blue line*) compared with free FAD (*red line*). The absorption spectrum of FsqB was recorded in sodium phosphate buffer, pH 7.6. *B*, the photoreduction of FsqB as a function of light irradiation, which causes a decrease of absorption at 460 nm and a sharp increase of absorption at 396 nm, indicating the formation of the *red* anionic semiquinone. After complete reduction to the flavin semiquinone, the oxidized flavin was regenerated within ∼70 min under aerobic conditions, *i.e.* reoxidation by dioxygen.

*FsqB* was expressed in *Escherichia coli* yielding 1 mg of FsqB from 1 g of pellet after purification by nickel–nitrilotriacetic acid affinity chromatography. For crystallization, the protein was further purified by means of size-exclusion chromatography. The UV-visible absorption spectrum of FsqB possesses the typical features of an FAD-containing protein with absorption maxima at 460 and 370 nm (Fig. 1*A*). The bathochromic shift of the long wavelength absorption maximum by ∼10 nm compared with free FAD is most likely due to the monocovalent attachment of the 8α-methyl group to cysteine residue 414. Similar UV-visible absorption spectra were previously reported for human dimethylglycine dehydrogenase (hDMGDH) (5) and *N*-methyl-tryptophan oxidase (6), which feature the same covalent linkage. The extinction coefficient of the FAD bound to FsqB was calculated to 12,350 m^−1^ cm^−1^. Consumption of *N*-methyl-dopa was investigated from pH 4 to 9 using an oxygen sensor to monitor the reaction yielding a bell-shaped profile with an optimum at pH 7.6. Photoreduction of FsqB led to the formation of a stable flavin semiquinone with an absorption maximum at 396 nm. Further reduction of the flavin semiquinone to the hydroquinone could not be achieved by light. The flavin semiquinone was reoxidized very slowly by molecular oxygen reaching completion after ∼70 min (Fig. 1*B*).

### Substrate screening

As part of the biosynthetic gene cluster in *A. fumigatus*, the cognate substrate of FsqB is bound to the “thiolation” domain of FsqF, and thus, this substrate is neither available nor convenient for the study of reaction kinetics (4). Moreover, we were interested to explore the reaction mechanism of FsqB using substrate analogs, as well as the potential use of the enzyme for biocatalytic applications. The five potential substrates tested with FsqB featured different hydroxylation at the aromatic ring (Table 1). Product analysis by HPLC–MS revealed that only *N*-methyl-dopa was oxidized to the corresponding isoquinoline derivative, whereas substrates lacking either the *para*- or *meta*-hydroxyl group, *i.e. N*-methyl-*meta*-tyrosine and *N*-methyl-tyrosine, yielded increased amounts of the demethylated product (24 and >99%, respectively; Table 1). On the other hand, adrenalone, epinephrine, and phenylephrine were unable to reduce the bound FAD cofactor of FsqB and thus are apparently not accepted as substrates.

**Table 1:**
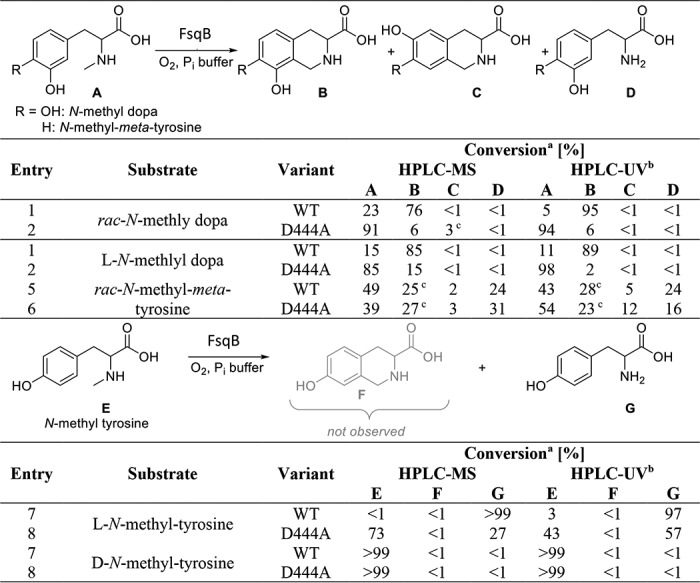
**Conversions of FsqB-catalyzed biotransformations** WT, wildtype enzyme.

*^a^* Conversions were determined by peak area integration of the corresponding MS or UV response.

*^b^* HPLC–UV chromatograms were recorded at 230-nm wavelength.

*^c^* No reference material was available. Peak identity is assumed in comparison with the other experiments and product formations and the *m/z* trace of the HPLC-MS peaks.

### Kinetic parameters of FsqB

**Figure 2.**
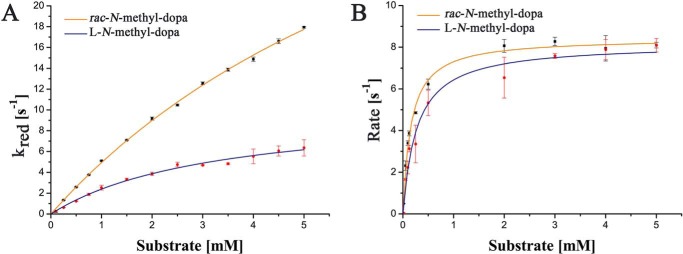
**Pre–steady-state (*A*) and steady-state kinetics (*B*).** The data for the racemic *N*-methyl-dopa and the l-stereoisomer are shown as *orange* and *blue lines*, respectively. The standard deviations were calculated based on three measurements for each substrate concentration.

Following product analysis, we also determined pre–steady-state parameters in the stopped-flow apparatus for *N*-methyl-dopa, *rac-N*-methyl-*meta*-tyrosine, and l-*N*-methyl-tyrosine. The highest rate of flavin reduction was observed with (racemic) *N*-methyl-dopa, whereas (racemic) *N*-methyl-*meta*-tyrosine and l-*N*-methyl-tyrosine were 10–25 times slower (the *k*_red_ values given in Table 2 were measured at a single fixed concentration of 2.5 mm). A more detailed analysis of the reduction of FsqB using *rac-N*-methyl-dopa or the l-stereoisomer yielded limiting rates of 51 ± 3 and 21 ± 3 s^−1^, as well as dissociation constants of 9.2 ± 0.7 and 8.0 ± 1.3 mm, respectively (Fig. 2*A* and Table 2). Thus, the limiting rate for l-*N*-methyl-dopa is only ∼2.5-fold lower than for the racemate, indicating that both stereoisomers are accepted by the enzyme. Furthermore, this result also suggests that the enzyme has a preference for d-*N*-methyl-dopa (commercially not available). To distinguish the rate of reduction for the d- and l-stereoisomer, we repeated the pre–steady-state measurements at a lower concentration of *rac-N*-methyl-dopa (*i.e.* 0.2 mm) and obtained a clear biphasic behavior with observed rates of 1.63 ± 0.04 and 0.61 ± 0.01 s^−1^, respectively, presumably reflecting the rate of reduction for the two stereoisomers in the racemic mixture. This 2.5-fold difference is similar to the difference observed for the racemate compared with the l-stereoisomer (Table 2), supporting our conclusion that the d-stereoisomer is a slightly better substrate than the l-stereoisomer. Notably, at substrate concentrations above 0.2 mm, the reaction showed essentially monophasic behavior because the fast-reacting enantiomer outcompetes the slower one. Interestingly, reduction of the FAD cofactor was not observed with d-*N*-methyl-tyrosine, suggesting that FsqB exhibits strict stereo-preference for the l-*N*-methyl-tyrosine stereoisomer (*S* configuration) in contrast to what was found with *N*-methyl-dopa.

**Table 2:** **Summary of pre–steady-state kinetic parameters for WT FsqB with different substrates** ND, not determined; NR, no reaction.

Substrate	*k*_red_	*k*_red_*^a^*	*K_D_*
	*s*^−*1*^	*s*^−*1*^	*mm*
*rac-N*-Methyl-dopa	51 ± 2.7	10.4 ± 0.1	9.2 ± 0.7
l-*N-*Methyl-dopa	21 ± 3	4.7 ± 0.01	8 ± 1.3
*rac-N-*Methyl-*meta*-tyrosine	6 ± 0.6	0.9 ± 0.008	15 ± 2
l-*N*-Methyl-tyrosine	ND*^b^*	0.6 ± 0.0004	ND*^b^*
d-*N*-Methyl-tyrosine	NR	NR	

*^a^* For comparison, the values of *k*_red_ were recorded at 2.5 mm substrate concentration because of the limited solubility of l-*N*-methyl-tyrosine.

*^b^* The rate of reaction increased linearly with increasing concentration preventing calculation of a *K_D_* and a limiting rate of reduction.

Steady-state kinetics were determined for *N*-methyl-dopa (racemate and the l-stereoisomer), l-*N*-methyl-tyrosine, and *rac-N*-methyl-*meta*-tyrosine using oxygen depletion to monitor enzyme turnover. As shown in Fig. 2*B*, enzyme activity was a function of substrate concentration reaching saturation at ≈8 s^−1^. Similarly, the *K_m_* for the racemate and the l-enantiomer of *N*-methyl-dopa differed only slightly, *i.e.* 0.16 ± 0.01 and 0.27 ± 0.02 mm, respectively. For l-*N*-methyl-tyrosine and *rac-N*-methyl-*meta*-tyrosine, saturation was reached at ≈4 and ≈3.5 s^−1^, respectively. The *K_m_* for these substrates was 2.1 ± 0.2 and 1.6 ± 0.6 mm, respectively. Table 3 summarizes the kinetic parameters for all substrates oxidized by FsqB. As judged by the resulting *k*_cat_/*K_m_* values, *N*-methyl-dopa (racemate and the l-enantiomere) is more efficiently oxidized by FsqB than l-*N*-methyl-tyrosine and *rac-N*-methyl-*meta*-tyrosine (Table 3).

**Table 3:** **Steady-state kinetic parameters for the studied substrates using WT FsqB**

Substrate	*K_m_*	*k*_cat_	*k*_cat_/*K_m_*
	*(mm)*	*s*^−*1*^	*mm*·*s*^−*1*^
*rac-N*-Methyl-dopa	0.16 ± 0.01	8.5 ± 0.1	53 ± 4
l-*N*-Methyl-dopa	0.27 ± 0.02	8.2 ± 0.2	30 ± 3
*rac-N*-Methyl-*meta*-tyrosine	1.6 ± 0.2	3.5 ± 0.1	2.18 ± 0.04
l-*N*-Methyl-tyrosine	2.1 ± 0.2	3.9 ± 0.2	1.91 ± 0.02
d-*N*-Methyl-tyrosine			

To determine the rate of reoxidation of FsqB, the enzyme was reduced with either (racemic) *N*-methyl-dopa, *N*-methyl-*meta*-tyrosine, or l-*N*-methyl-tyrosine under anoxic conditions and reacted with oxygen-containing buffer in the stopped-flow apparatus. This yielded rates for the reoxidation of (20.5 ± 0.9) × 10^3^, (4.5 ± 0.2) × 10^3^, and (4.2 ± 0.1) × 10^3^ m^−1^ s^−1^, respectively.

### X-ray crystallographic structure of FsqB

**Figure 3.**
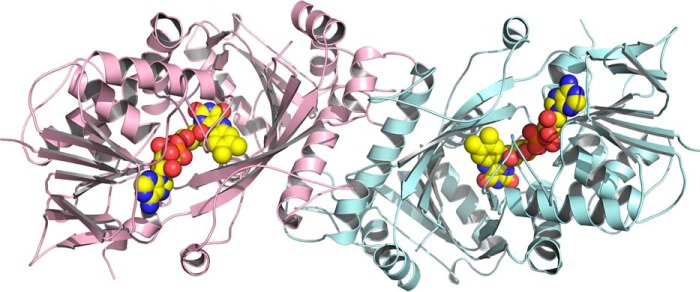
**Cartoon representation of the FsqB dimer present in the crystal as predicted by the EBI-Pisa server (7) looking down the crystallographic 2-fold symmetry axis.** The two protomers are colored *pink* and *light blue*, respectively. The bound flavin cofactors are depicted as *spheres*.

We determined the crystal structure of FsqB to a resolution of 2.6 Å (Table 4). The asymmetric unit of the hexagonal crystals contained only one protein chain, but an analysis using the EBI-Pisa server (7) indicated the existence of a dimeric assembly involving a symmetry-related molecule generated by a crystallographic 2-fold symmetry axis (Fig. 3). In this assembly, ∼2900 Å^2^ of solvent-accessible surface are buried on each protomer, predicting that this dimer should also be formed and stable in solution.

**Table 4:** **Crystal structure determination: data collection and refinement statistics** Statistics for the highest-resolution shell are shown in parentheses.

	FsqB
Wavelength (Å)	0.95
Resolution range (Å)	45.06–2.60 (2.69–2.60)
Space group	*P*6_5_22
Unit cell (Å, °)	90.12, 90.12, 266.28, 90, 90, 120
Total reflections	316,554 (28,058)
Unique reflections	20,505 (1862)
Multiplicity	15.4 (15.1)
Completeness (%)	98.9 (92.7)
<*I*/σ(*I*)>	24.20 (2.66)
Wilson B-factor	65.53
*R*_merge_	0.0827 (0.7854)
*R*_meas_	0.0856 (0.8121)
*R*_pim_	0.0218 (0.2039)
CC½	1 (0.95)
CC*	1 (0.99)
Reflections used in refinement	20,430 (1856)
Reflections used for *R*_free_	1022 (93)
*R*_work_	0.2123 (0.2647)
*R*_free_	0.2432 (0.3362)
CC_work_	0.95 (0.93)
CC_free_	0.96 (0.82)

Number of non-H atoms	3945
Macromolecule	3856
Ligands/cofactors	53
Solvent	36
RMSD bonds (Å)	0.006
RMSD bond angles (°)	0.83
Ramachandran favoured (%)	95.73
Ramachandran allowed (%)	4.27
Ramachandran outliers (%)	0.00
Rotamer outliers (%)	0.00
Clashscore	7.89
Average B-factors (Å^2^)	71.43
Macromolecules	71.68
Ligands/cofactors	60.67
Solvent	61.05

**Figure 4.**
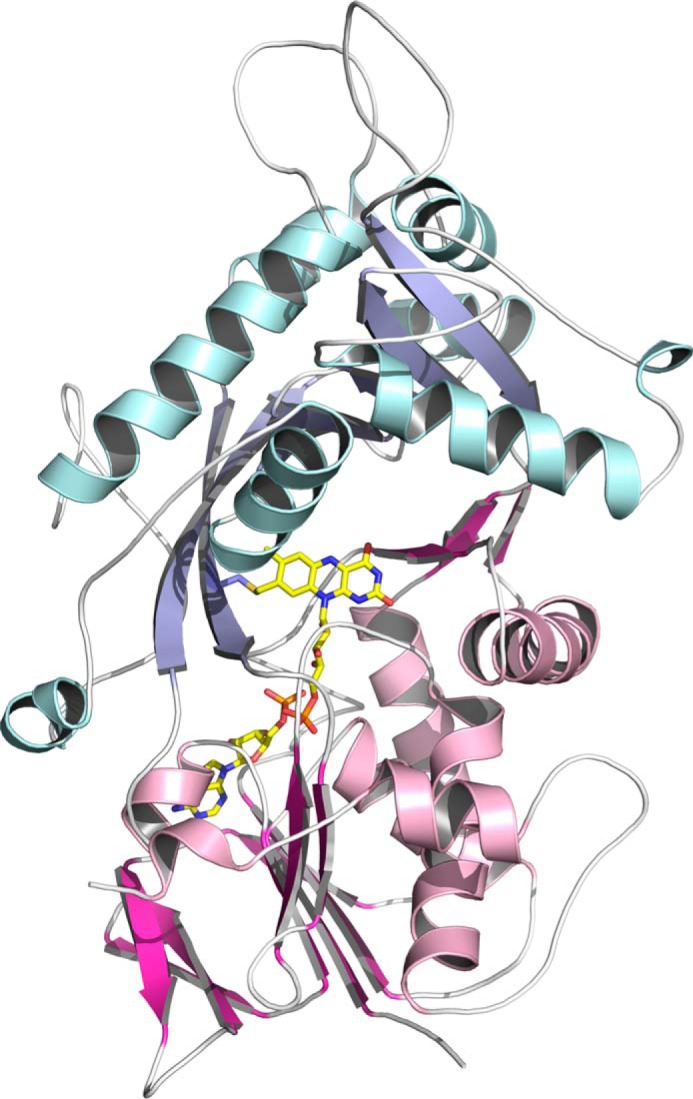
**Cartoon representation of the structure of the FsqB protomer.** The FAD-binding domain is shown in *magenta* (β-strands) and *pink* (α-helices), and the substrate-binding domain is shown in *light blue* (β-strands) and *light cyan* (α-helices). The covalently bound FAD is depicted as *sticks* (*yellow*) together with the tethering amino acid Cys-414.

The FsqB protomer consists of two discontinuous domains (Fig. 4), an FAD-binding domain (residues 1–97, 183–257, and 419–496) and a substrate-binding domain (residues 98–182 and 259–418). The FAD-binding domain exhibits a three-layer ββα-fold with the diphosphate group of FAD bound at the C-terminal edge of a mostly parallel, central β-sheet. The most prominent feature of the substrate-binding domain is a seven-stranded, mostly antiparallel β-sheet, which spans across the bound flavin. The two N-terminal strands of this sheet wrap around the dimethyl-benzene part of the isoalloxazine ring system and provide the amino acid residue Cys-414, which is covalently bound to the 8α-methyl group of the FAD cofactor. The substrate-binding domain is also responsible for dimer formation. The central portion of the interface between the two protomers is formed by a long α-helix situated on top of the seven-stranded β-sheet as well as by surrounding loop regions (Fig. 4).

**Figure 5.**
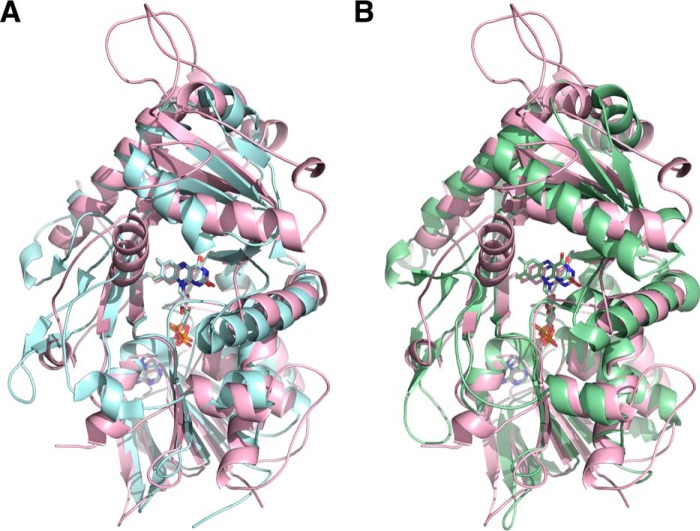
**Cartoon representations of the FsqB structure (*pink*) superimposed on the structure of fructosamine oxidase from *A. fumigatus* (*A*, *cyan* (8)) and the fructosyl peptide oxidase from *E. terrenum* (*B*, *green* (9)).**

A search for structurally similar proteins using the PDBeFold server yielded the fructosamine oxidase from *A. fumigatus* (PDB entry 4WCT) (8) and the fructosyl peptide oxidase from *Eupenicillium terrenum* (PDB entry 4RSL) (9) as the closest structural homologs with *Q* scores greater than 0.35 and root-mean-square deviations (RMSDs) of ∼2 Å. A superposition of FsqB with these two structures is shown in Fig. 5 highlighting the fold similarities.

**Figure 6.**
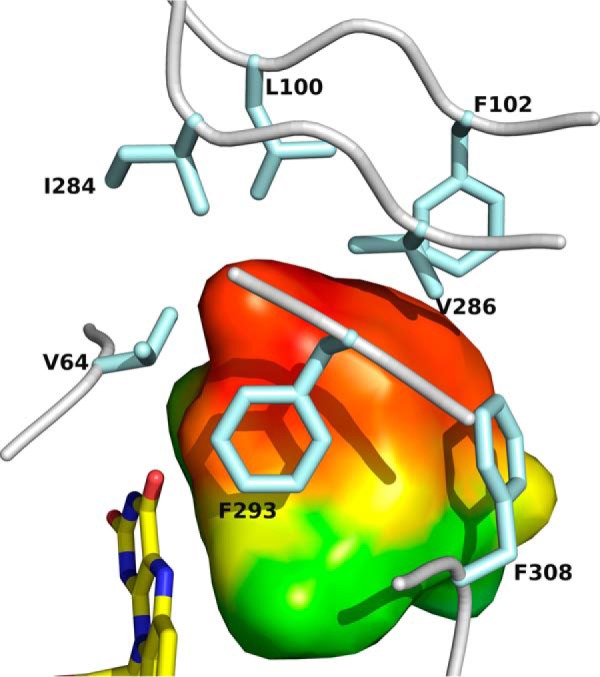
**Close-up view of the active site cavity in the vicinity of the pyrimidine ring of the flavin cofactor.** The cavity volume is depicted as a surface representation colored by hydrophobicity (*red*, hydrophobic; *green*, hydrophilic). Cavity points were calculated and annotated using the program CASoX (G. Steinkellner, unpublished data). The FAD cofactor and the side chains lining this part of the active site cavity are shown as *sticks* (29).

The active site of FsqB is located at the interface between the FAD binding and the substrate-binding domain at the core of the protein and is connected to the solvent by a long and rather broad tunnel. The covalently bound isoalloxazine ring is surrounded on both sides by mostly polar residues, but a hydrophobic pocket lined by Val-64, Leu-100, Phe-102, Ile-284, Val-286, Phe-293, and Phe-308 is situated on the *re*-side of the cofactor (Fig. 6).

### Modeling of the substrate complex

**Figure 7.**
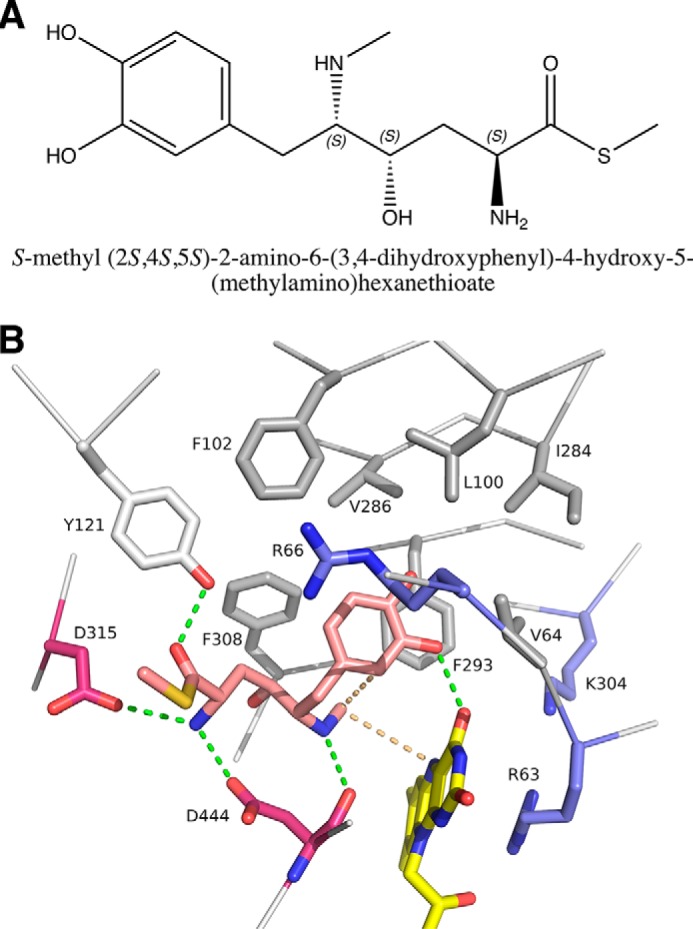
*A*, chemical structure of the truncated, natural substrate used in the docking calculations. *B*, close-up view of the active site of FsqB in the modeled complex with the truncated, natural substrate (*pink*). The flavin cofactor is shown in *yellow*. Positively charged residues in vicinity are shown in *blue*, and negatively charged residues are in *magenta*. Residues forming the hydrophobic pocket are shown in *gray. Green dashed lines* depict hydrogen bonding interactions. The interactions between the *N*-methyl group of the substrate and N5 and the aromatic moiety are shown as *light orange, dashed lines*.

Crystals of FsqB were soaked with various, potential substrates to determine the structure of an enzyme–substrate complex. However, either the treated crystals did not diffract to high resolution, or the resulting difference electron density was not clear enough to be interpreted as a bound substrate. Therefore, we resorted to molecular docking simulations to obtain a model of such a complex. For these calculations, we chose a truncated variant of the natural substrate, *S*-methyl (2*S*,4*S*,5*S*)-2-amino-6-(3,4-dihydroxyphenyl)-4-hydroxy-5-(methylamino)-hexanethioate (Fig. 7*A*). A number of active site residues, especially around the hydrophobic pocket (Fig. 6) were treated as flexible (see “Experimental procedures”). Because of the high number of the resulting internal degrees of freedom, we obtained many different docking poses and analyzed them with respect to mechanistic plausibility, *i.e.* we selected poses where the *N*-methyl group was close (<4 Å) to the N5 atom of the FAD and was appropriately positioned to form a C–C bond with the dihydroxyphenyl moiety to form the isoquinoline ring. Of ∼20 different binding modes, only one fulfilled these requirements (Fig. 7*B*).

In this model structure, the dihydroxyphenyl group is bound in the hydrophobic pocket and also interacts with the side chain of Arg-66. In addition, the 3-OH group forms a hydrogen bond with the C4=O of the flavin cofactor. Furthermore, Arg-63 and Lys-304 are near the isoalloxazine ring of the FAD and the dihydroxyphenyl group of the substrate. The resulting positive electrostatic potential could facilitate the deprotonation of the substrate at the 3-OH group of the catechol moiety or favor the binding of an already deprotonated substrate. In the crystal structure, there is also an ordered water molecule located right between O4 and the amino group of Lys-304, which may be displaced upon substrate binding.

The carbon atom of the *N*-methyl group is close to N5 of the flavin (required for hydride transfer) and is also appropriately positioned to attack C2 of the aromatic ring (3.5 Å). The corresponding nitrogen atom is in hydrogen bonding position to the main chain carbonyl oxygen of Asp-444. The nitrogen atom at the 2 position of the substrate on the other hand forms salt bridges to the side-chain carboxylates of Asp-315 and Asp-444. Thus, these two residues are very likely primarily involved in substrate binding and orientation.

The methyl-thioester group of the bound ligand is oriented toward the entrance of the active site tunnel. This binding mode is therefore compatible with a longer chain (*e.g.* as in a CoA derivative) being attached to the substrate.

**Figure 8.**
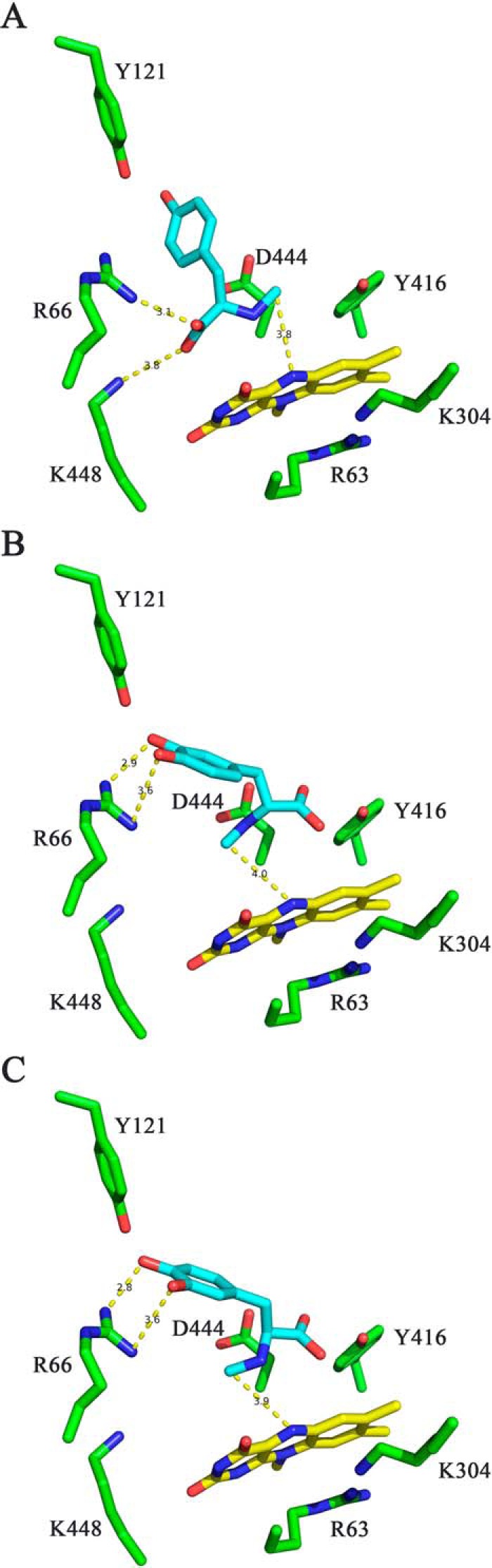
**Stick representations of the suggested binding scenarios for l-*N*-methyl-tyrosine (*A*) and the two stereoisomers of *N*-methyl-dopa (*B* and C).** Amino acid side chains near the binding site are shown in *green*. The isoalloxazine ring of FAD is shown in *yellow*. The docked substrates are shown in *cyan*. The distances of the carboxylate group of l-*N*-methyl-tyrosine to Arg-66 and Lys-448 and of the catechol to Arg-66 are shown as *yellow dashed lines. A–C* show a model for l-*N*-methyl-tyrosine, d-*N*-methyl-dopa, and l-*N*-methyl-dopa bound to the active site of FsqB, respectively.

In addition, we performed molecular docking simulations with the substrates used in our study, *i.e. N*-methyl-dopa and l-*N*-methyl-tyrosine. Because of the reduced spatial requirements of these substrates, we found two distinct binding modes that are different to the binding pose for the (truncated) natural substrate. As shown in Fig. 8*A*, l-*N*-methyl-tyrosine binds with the carboxylate group oriented toward the pair of positively charged amino acids (Arg-66 and Lys-448) and the phenolate interacts with Tyr-121. This pose also places the *N*-methyl group near the N5 of the isoalloxazine ring, thereby allowing the transfer of a hydride. In contrast to the binding pose of l-*N*-methyl-tyrosine, the two stereoisomers of *N*-methyl-dopa may bind in a different orientation with the catechol-ring oriented toward Arg-66 and Tyr-121. In both poses, the *N*-methyl group is placed above the N5 to enable hydride transfer (Fig. 8, *B* and *C*). In all three docking poses, the amino-acid side chain of Asp-444 is within 3–4 Å of the *N*-methyl group.

### Site-directed mutagenesis of active site amino acid residues

Based on the crystal structure of FsqB and the molecular docking of the natural substrate, we generated several FsqB variants to probe the involvement of amino acids in catalysis using *N*-methyl-dopa as the substrate. With the exception of the K448A variant, all variants exhibited a similar thermal melting point of ≈55 °C and a similar CD spectrum, indicating that the proteins were properly folded and possessed a similar stability. Replacement of the three basic residues Arg-63, Arg-66, and Lys-304 severely compromised enzymatic activity, and the R63M and K304A variants showed only residual activity amounting to 0.3 and 3% of the activity of the WT enzyme (Table 5). Similarly, replacement of Tyr-121 to phenylalanine resulted in a substantial loss of activity (2% residual activity). Very surprisingly, the D444A variant substantially reduced enzymatic activity of FsqB (0.03% residual activity), suggesting an important role in catalysis. Interestingly, the K304A variant exhibited a very slow rate of reoxidation of 200 ± 40 m^−1^ s^−1^ and thus is apparently involved in the oxidative half-reaction, *i.e.* oxidation of the reduced FAD by dioxygen. The role of Lys-448 for covalent flavinylation was probed by replacing this residues with alanine. The resulting K448A variant was completely devoid of FAD, confirming earlier findings for MSOX that this residue plays an essential role for establishing the covalent bond between the 8α-methyl group and the corresponding cysteine residue (10). From the results of the mutagenesis study (Table 5), we suggest that the positively charged side chains of residues Arg-66 and Lys-448 are important for substrate binding, whereas the carboxyl group of the side chain of Asp-444 activates the substrate by proton abstraction from the amino group of the substrate. On the other hand, Arg-63 located on the *si* face stabilizes the negatively charged flavin cofactor after hydride transfer from the substrate. Finally, Lys-304 was identified as a central player in the reoxidation of the reduced flavin by dioxygen.

**Table 5:** **Summary of pre–steady-state kinetic parameters for FsqB variants with *rac-N*-methyl-dopa** ND, not determined.

Enzyme	*k*_obs_*^a^*
	*s*^−*1*^
R63M	0.027 ± 0.003
R66M	ND
Y121F	0.24 ± 0.03
K304A	0.3 ± 0.03
Y416F	3.4 ± 0.02
D444A	0.0036 ± 0.0004

*^a^* The values of *k*_obs_ were recorded for comparison at 2.5 mm substrate concentration because of the limited solubility of l-*N*-methyl-tyrosine.

## Discussion

Based on the sequence similarity to members of the amine oxidase family, *e.g.* MSOX and MTOX, it was recently suggested that FsqB shares the topology of this protein family. The X-ray crystal structure of FsqB reported here supports this assumption. This family of FAD-dependent oxidases was extensively studied in terms of the mechanism of oxidation. Among the discussed mechanisms, a hydride transfer mechanism, entailing the transfer of a hydride from the *N*-methyl group to the N5 position of the isoalloxazine ring, appears to be the most favorable (11, 12). In addition, recent fragment molecular orbital and mixed quantum mechanics/molecular mechanics calculations have concluded that a hydride transfer mechanism is the most likely scenario (12, 13). Therefore, we assumed that the oxidative cyclization catalyzed by FsqB might be initiated by a hydride transfer, followed by the nucleophilic attack of the catechol moiety of the substrate to yield the isoquinoline product because the electron-rich catechol moiety can be easily deprotonated at the 3-OH (p*K_a_* = ∼9). In fact, docking of the proposed natural substrate to the active site of FsqB suggested that the *N*-methyl group is positioned on the *re*-side of the isoalloxazine ring close to the N5 in accordance with the steric requirements for a hydride transfer (Fig. 7*B*). Although this docking pose is compatible with a hydride transfer mechanism, it fails to explain the effects on catalysis observed in some of the generated FsqB variants, most notably the R66M and D444A variants. Thus, we hypothesized that the substrates tested in our study adopt a different binding pose as suggested for the natural substrate. In fact, docking simulations with l-*N*-methyl-tyrosine and *N*-methyl-dopa showed that both of these substrates can bind in different orientations as compared with the natural substrate. In the case of l-*N*-methyl-tyrosine, the carboxylate group interacts with the side chains of Arg-66 and Lys-448, and the phenolate ring points toward Tyr-121 (Fig. 8*A*). This binding mode is very similar to the binding of substrates in other members of the amine oxidase family (PF01266), notably MTOX, MSOX, and NikD (14–16) and is clearly also possible in FsqB because of the conservation of this pair of positively charged amino acid residues. In addition, this binding mode also provides a role for the side chain of Asp-444 because the carboxylate is located near the *N*-methyl group of the substrate and may act as an active site base to promote the transfer of a hydride to the N5 of the isoalloxazine ring, thus accounting for the large effect on catalysis observed in the D444A variant. Interestingly, docking of the two stereoisomers of *N*-methyl-dopa suggested a different binding mode with the catechol ring moiety engaging in a salt bridge with the guanidinium group of Arg-66 and the carboxylate group pointing toward the side chain of Tyr-416 (Fig. 8, *B* and *C*). Again, in these binding poses, the side chain of Asp-444 is in close proximity to the *N*-methyl group and may participate in catalysis as an active site base. Overall, the putative binding poses for *N*-methyl-dopa are in agreement with effects observed with the generated FsqB variants: very low activity for the R66M and D444A variants, a less pronounced effect for the Y121F variant, and a very small effect in the case of the Y416F variant. It is also noteworthy that both stereoisomers are productively converted to the isoquinoline product with similar kinetic rates, indicating that both poses are conducive for the ring closure reaction. In this context, it is very interesting to note that the distinct binding poses for l-*N*-methyl-tyrosine and *N*-methyl-dopa also provide a rationale for the finding that FsqB accepts only the l-stereoisomer but not the d-stereoisomer of *N*-methyl-tyrosine, whereas both stereoisomers of *N*-methyl-dopa are accepted, albeit with slightly different kinetic rates (Table 2): l-*N*-methyl-tyrosine is anchored to Arg-66 and Lys-448 via its carboxylate group fixing the chiral carbon in the position shown in Fig. 8*A*, and thus, binding of the d-stereoisomer would require the rotation of the substrate (by ∼180°) above the *re*-side of the isoalloxazine ring, which is very unlikely because there are no residues “on the other side” to interact with the carboxylate group. In contrast, a similar rotation is not required for *N*-methyl-dopa because the substrate is anchored to Arg-66 by the catechol moiety and not the carboxylate group, and therefore it is sufficient to allow rotation of the C–C bond connecting the chiral carbon atom to the β-carbon atom. In keeping with this interpretation, *N*-methyl-dopa (and the l-enantiomer) is more efficiently consumed by FsqB than *N*-methyl-tyrosine and *N*-methyl-*meta*-tyrosine (20-fold difference in *k*_cat_/*K_m_*; Table 3), suggesting that positioning the catecholate moiety toward Arg-66 is the preferred binding mode for these substrate analogs. In summary, our results suggest that the observed stereospecificity for *N*-methyl-tyrosine (and *N*-methyl-*meta*-tyrosine) is the result of the specific binding mode, whereas the different binding mode of *N*-methyl-dopa in combination with the active site plasticity of FsqB accommodates both stereoisomers. Currently, efforts to obtain a crystallographic structure of d- and l-*N*-methyl-tyrosine bound to FsqB are underway to obtain more structural insights into the parameters that control substrate binding and oxidation in particular with regard to the observed stereospecificity.

**Scheme 1.**
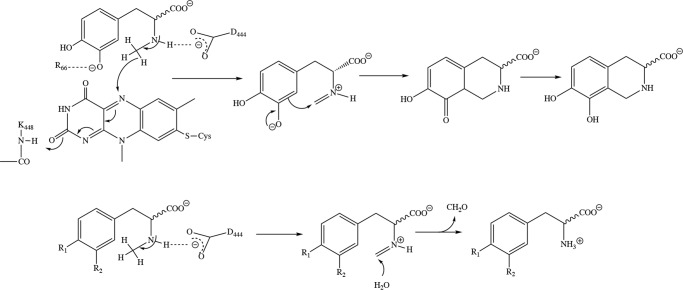
**Proposed reaction mechanism for the ring closure reaction with *N*-methyl-dopa (*top panel*) and for the demethylation via hydrolysis of the imine occurring with *N*-methyl-tyrosine (R_1_=OH, R_2_=H) and partially with *N*-methyl-*meta*-tyrosine (R_1_=H, R_2_=OH) (*bottom panel*).**

The demethylation observed with *rac-N*-methyl-*meta*-tyrosine and l-*N*-methyl-tyrosine (Table 1) can be explained by the lack of the *meta*-hydroxyl group in the case of the latter and a lower efficiency for the ring closure reaction in the binding mode adopted by these substrates (Fig. 8*A*). The reduction of the flavin by the substrate results in the formation of an imine that is subsequently attacked by the phenolate ring (Scheme 1). However, this reaction is not possible in l-*N*-methyl-tyrosine, and thus, the imine intermediate will hydrolyze to the demethylated product, *i.e.* l-tyrosine. The lack of the *para*-hydroxyl group, on the other hand, may also slow down the ring closure step and therefore lead to an efficient competition by hydrolysis yielding a mixture of cyclized and demethylated product in the case of *rac-N*-methyl-*meta*-tyrosine as substrate.

The oxidation of reduced FAD by molecular oxygen in the family of amine oxidases has been the subject of several detailed studies revealing the importance of a positive charge near the site of reoxidation, the C4a position of the isoalloxazine ring (17, 18). In accordance with these studies, we have identified Lys-304 as a central amino acid residue for the oxidative half-reaction, because the K304A variant exhibited a more than 100-fold decreased rate. The importance of this residue for the reactivity of the reduced FAD with oxygen was also observed with the related MSOX (19); thus, our finding adds further support to the current concept of oxygen activation in flavoprotein amine oxidases. We have also observed different rates for the reoxidation of reduced FsqB depending on the substrate used for the reduction, *i.e.* a fast reaction with *N*-methyl-dopa and 5-fold lower reaction with l-*N*-methyl-tyrosine and *rac-N*-methyl-*meta*-tyrosine. In the case of MSOX, it was proposed that oxygen reacts with the enzyme–product complex (20), and thus, the observed differences in the kinetic rates may reflect the differences of the enzyme–product complex obtained with l-*N*-methyl-tyrosine (only demethylated product, *i.e.* tyrosine) and *rac-N*-methyl-*meta*-tyrosine (demethylated and isoquinoline product) on the one hand and *N*-methyl-dopa (only isquinoline product) on the other hand.

In summary, our study has shown that FsqB is a member of the family of amine oxidases, but in contrast to previously characterized members of this family, it combines the oxidation of a *N*-methyl group with the generation of a heterocyclic ring system. Although FsqB is the first characterized representative of this group of “oxidative cyclases,” database searches using the FsqB sequence revealed many more putative enzymes that may catalyze a similar ring closure reaction in natural product biosynthesis, in particular in fungi. Also, the plasticity of the active site of FsqB, and potentially of other yet uncharacterized homologs, may encourage further developments to improve the substrate scope of the enzyme for biocatalytic applications.

## Experimental procedures

### Chemicals

The gene encoding FsqB was synthesized by Invitrogen Life Technologies. Restriction enzymes, ligases, and Phusion DNA polymerase were from Thermo Fisher Scientific. Prepacked nickel-Sepharose fast flow columns were from GE Healthcare. Solvents, media components, and buffer salts were from Carl Roth GmbH (Karlsruhe, Germany). Enzyme substrates and salt-free purified oligonucleotides for site-directed mutagenesis were from Sigma–Aldrich. *Rac-N*-methyl-dopa, l-*N*-methyl-dopa, and *rac-N*-methyl-*meta*-tyrosine were synthesized as described in the supporting information.

### Molecular cloning

On ordering, the gene sequence of FsqB (Afu6g03440) was codon-optimized for expression in *E. coli* and an octahistidine tag was added to the C terminus. Additionally, the gene was flanked with an NdeI and a NotI restriction site at the 5′ and 3′ ends, respectively. The DNA string was cloned into a shuttle vector (pJET) and transformed into *E. coli* Top 10 cells (Stratagene) for strain preservation and plasmid propagation. After digestion with NdeI and NotI, the gene was cloned into the *E. coli* expression vector pET21a, conferring ampicillin resistance. Correct insertion of the gene sequence was verified by sequencing, before transforming the plasmid into *E. coli* BL21 Star (Stratagene) cells for expression.

### Protein expression and purification

Protein production and purification was adapted from Baccile *et al.* (4). FsqB was expressed in shake flasks in an HT Multitron standard shaking system (Infors AG, Basel, Switzerland). Main cultures were inoculated to an *A*_600_ of ∼0.1, by adding an overnight culture to fresh LB medium containing 100 μg/ml ampicillin, and incubated at 37 °C and 140 rpm until an optical density of 0.7 was reached. Then production of FsqB was induced by adding 300 μm isopropyl β-d-thiogalactopyranoside, before incubating the cultures at 18 °C for 15 h to maximize the protein yield. The cells were harvested by centrifugation at 5,000 × *g* for 15 min and stored at −20 °C until further use. Protein purification was carried out under low light conditions at 4 °C. To purify the enzyme, the pellet was suspended in lysis buffer (100 mm phosphate/NaOH, 150 mm sodium chloride, 10 mm imidazole, pH 7.6, containing 1 mm phenylmethylsulfonyl fluoride and 0.1 mg/ml lysozyme) and sonicated using a Labsonic U sonication probe (B. Braun Biotech, Berlin, Germany) for 10 min. The lysate was centrifuged at 38,500 × *g* for 60 min, and the supernatant was filtered through a filter paper. The clear supernatant was loaded onto a nickel–nitrilotriacetic acid–Sepharose fast flow column (GE Healthcare) prewashed with lysis buffer without phenylmethylsulfonyl fluoride and lysozyme. The column was washed with 10 column volumes of washing buffer (100 mm phosphate/NaOH, 150 mm NaCl, 30 mm imidazole, pH 7.6), before FsqB was eluted with elution buffer (100 mm phosphate/NaOH, 150 mm NaCl, 150 mm imidazole, pH 7.6). The eluted protein was dialyzed against 100 mm phosphate/NaOH, 150 mm NaCl, pH 7.6 (storage buffer) overnight. Then the protein was concentrated in an Amicon Ultra-15 centrifugal filter with a 30-kDa cutoff (Merck–Millipore). For protein crystallization FsqB was further purified by size-exclusion chromatography using a Superdex 200 prep grade XK 16/60 column (GE Healthcare), equilibrated with storage buffer, and attached to an ÄKTA FPLC system (GE Healthcare) at 4 °C. Protein purity was monitored by SDS-PAGE with 12.5% separation gel (FsqB molecular mass is 55 kDa).

### UV-visible absorption spectroscopy and calculation of the extinction coefficient

A Specord 210 spectrophotometer (Analytik Jena, Jena, Germany) was used for UV-visible absorption spectroscopy. Concentrations of purified enzyme samples were determined according to the absorption of bound FAD at 450 nm. The molar extinction coefficient for FsqB was determined as described in Ref. 21.

### Protein thermal stability

The thermal stability of FsqB was assessed by recording the change in fluorescence caused by the release of the FAD cofactor as an intrinsic probe to monitor protein stability and folding in a ThermoFAD assay (22). The measurements were carried out in triplicate with an FX Connect real-time PCR system (Bio-Rad) in a 25-μl mixture of 100 mm phosphate/NaOH, pH 7.6, containing 150 mm NaCl, and 3 mg/ml protein. The starting temperature was 20 °C for 5 min and then it was increased at a rate of 0.5 °C/min to 95 °C. The CFX Manager 3.0 software was used to determine the melting temperatures for WT FsqB and the variants generated by site-directed mutagenesis. Furthermore, ThermoFAD was used to assess the impact of substrates and substrate analogs on the melting point of FsqB.

### CD spectroscopy

CD measurements were performed with a Jasco J715 (JASCO Inst., Gross-Umstadt, Germany) spectropolarimeter using a 0.01-cm water-jacket cylindrical cell. The far-UV spectra were recorded at 20 °C from 190 to 260 nm as an average of three scans. A protein concentration of 0.2 mg/ml was used for all measurements.

### Product identification

The products generated from the conversion of substrates by FsqB were analyzed by HPLC–MS. For HPLC–MS analysis, 5 mm of the potential substrate were incubated with 5 μm FsqB in 1 ml of 10 mm phosphate/NaOH buffer, pH 7.6, for 1 h at 25 °C with shaking at 600 rpm. Then the reactions were quenched with 200 μl of 0.2 m guanidine hydrochloride, and the samples were spun down at 10,000 × *g* for 10 min to remove denatured protein. The supernatant was then analyzed by HPLC–MS. Low resolution mass spectra were recorded with an Agilent Technologies 6120 Quadrupole LC/MS detector in combination with an Agilent Technologies 1260 Infinity HPLC system, equipped with a Kinetex 2.6 μ C-18 100A column (50 × 4.6 mm, 2.6 micron). Water/acetonitrile (+0.1 vol % of formic acid) was used as eluent. HPLC–UV analysis was carried out with a Shimadzu HPLC system (DGU-20A (degasser), LC-20A (pump), SIL-20A (autosampler), CTO-20AC (column oven), SPD-M20A (detector), and CBM-20AC (controller)) with water/acetonitrile (+0.1 vol % of TFA) as eluent using a Phenomenex Luna 5 μ C18 100A column. NMR spectra were recorded with a Bruker NMR unit at 300 (^1^H) and 75 (^13^C) MHz, shifts are given in ppm, and coupling constants (*J*) are given in Hz.

### Crystallization and crystal structure determination

Screening for crystallization conditions was performed with an Oryx8 robot (Douglas Instruments, Berkshire, UK) using the following commercially available screens: JCSG+ MD1–37, Morpheus Screen MD1–46 (Molecular Dimensions, Suffolk, UK), and Index HT HR2–144 (Hampton Research, Aliso Viejo, CA). Trials were set up in 96-well Swissci plates (Molecular Dimensions) using the sitting-drop vapor-diffusion method. Drops of 1 μl were pipetted with a 1:1 ratio of protein (concentrations of 10, 20, and 30 mg/ml in 100 mm phosphate/NaOH, pH 7.8, and 150 mm NaCl) and screening solution. The crystallization plates were incubated at 289 K.

Initial crystals of FsqB were obtained within 2 weeks in a crystallization condition containing 0.26 m ammonium sulfate and 0.2 m lithium sulfate in 0.1 m Tris/HCl, pH 8.5. Most of the crystals showed anisotropic diffraction to only 3.5–4 Å resolution. The best native crystal diffracted to 2.6 Å, and this data set was used for structure solution. Crystals were also soaked with different potential substrates of FsqB by adding a 0.125 mm solution of the respective compound directly to the crystallization drop using a cryo-loop. The soaked crystals were flash-cooled after different soaking times (15 s to 5 min) without the use of an additional cryoprotectant.

The crystals were screened and diffraction data were collected at 100 K at the Synchrotron sources Elettra (Trieste, Italy; Beamline XRDI), ESRF (Grenoble, France; Beamlines ID23-1, ID23-2, ID30A-3, and ID30B), and PETRA III (Hamburg, Germany; Beamlines P11 and P14). The data were processed and scaled using the XDS program package (23). The structure was solved by a combination of molecular replacement using the program Phaser (24), extensive manual rebuilding in Coot (25), and refinement using the PHENIX software suite (26). Molecular replacement was performed using the structure of fructosamine oxidase from *A. fumigatus* (PDB entry 3DJE) as search template. A randomly chosen set of 5% of the reflections was not used in the refinement but was set aside for *R*_free_ calculations (27). The stereochemistry and geometry of the resulting model were analyzed using the program MolProbity (28). Data collection and processing statistics are summarized in Table 4. Atomic coordinates and structure factors have been deposited in the Protein Data Bank as entry 6GG2. Visualization of structures (Figs. 3–8) was done using the program PyMOL (29).

### Site-directed mutagenesis

The pET21a-FsqB WT construct was used as a template in the PCR-based mutagenesis. Primers were designed (Table S1 in supporting information) to introduce the desired mutations in the codons pertaining to the targeted amino acids (30). The validity of the generated variants was confirmed by sequencing.

### Molecular docking

The natural substrate (truncated to the methyl-thioester; Fig. 7) was docked into the active site using the program ADFR (31). Protein and ligand structures were prepared using the program Maestro from the Schrödinger package (32). 52 independent docking runs employing the genetic-algorithm optimizer implemented in ADFR were executed with a maximum number of 2.5 million energy evaluations. During these simulations the side chains of the following residues were treated as flexible: Val-64, Leu-100, Ile-284, Val-286, Phe-293, and Val-295. The resulting docking poses were clustered using a maximum RMSD of 2 Å. The lowest-energy docking pose from each cluster was then visually analyzed with respect to the distance of the *N*-methyl group to the N5 of the Flavin, as well as regarding the conformation assumed to be necessary for promoting ring closure. Selected complex structures were further optimized using the program Prime from the Schrödinger package (32). In these calculations, all amino acid residues within 10 Å of the bound ligand were allowed to move, and an implicit solvent model was employed.

We have also performed docking simulations with l-*N*-methyl-tyrosine and the two stereoisomers of *N*-methyl-dopa using the Autodock Vina Plugin (33) of the Yasara structure suite (version 17.3.30, Yasara Biosciences (34)). Docking was performed with the receptor kept rigid, whereas the ligand was flexible and with a docking cell lining the cavity of the active site of FsqB. For each ligand, 250 independent docking runs were performed, and the resulting poses were clustered with an RMSD cutoff of 1 Å. Docking poses representing the lowest energy clusters were inspected visually.

### Determination of pH optimum and steady-state kinetics

To evaluate the catalytic activity and the pH optimum of FsqB, the oxygen consumption in the presence of various concentrations of *N*-methyl-dopa was recorded in triplicate. The measurements were performed utilizing a retractable needle-type oxygen sensor (Type OXR50-UHS) (Pyro Science GmbH, Aachen, Germany (35)). The pH optimum was determined by monitoring the enzymatic conversion of *N*-methyl-dopa (2 mm) as a substrate for FsqB in 0.1 m citrate/NaOH buffer from pH 4.0 to pH 5.5, in 0.1 m Hepes/NaOH from pH 6.0 to 7.0, and 0.1 m Tris/HCl from pH 7.0 to 8.0 and in 0.1 m borate/HCl from pH 8.0 to 9.0.

Kinetic assays were performed in 100 mm Tris/HCl, 150 mm NaCl buffer, pH 7.6, saturated with ambient air at 25 °C with enzyme concentrations of 10 μm in an air-tight cell. Experiments were started by the addition of FsqB to the sample cell containing 0–5 mm *N*-methyl-dopa. The oxygen consumption was monitored for 1 min, and the initial velocity was determined in the linear part of the measurement (typically 12 s). The *K_m_* and *k*_cat_ values were determined by employing the ORIGIN 8.6 software (OriginLab Corp.).

### Pre–steady-state kinetics and determination of k_ox_

Pre–steady-state reaction kinetics were measured anaerobically with a Hi-Tech stopped flow instrument (SF-61DX2; TgK Scientific Limited, Bradford-on-Avon, UK) in a glove box (Belle Technology, Weymouth, UK) at 25 °C. The reductive rates of enzyme-bound FAD were determined using a final concentration of 25 μm FsqB in 100 mm phosphate/NaOH buffer, 150 mm NaCl, pH 7.6. Enzyme was mixed with various concentrations of substrates (0–10 mm) or l-*N*-methyl-tyrosine (0–5 mm) dissolved in 100 mm phosphate buffer, 150 mm NaCl, pH 7.6, and spectral changes were detected with a Kineta-ScanT diode array detector (MG-6560; TgK Scientific Limited) and subsequently analyzed at 460 nm. Flavin reduction was monitored at each substrate concentration in triplicate, and the observed rate constants (*k*_obs_) for different substrate concentrations were calculated using an exponential fitting function in the KINETIC STUDIO software (TgK Scientific Limited). By plotting these observed rate constants as a function of the respective substrate concentrations, the reductive rates (*k*_red_), as well as dissociation constants (*K_D_*), could be determined by employing ORIGIN 8.6 software (OriginLab Corp.). The oxidative rates (*k*_ox_) were determined three times at a final oxygen concentration of 10.5% (135 μm oxygen), by mixing substrate-reduced FsqB with air saturated buffer (100 mm phosphate/NaOH, 150 mm NaCl, pH 7.6). By dividing the observed rate constants by the amount of oxygen dissolved in the buffer (final concentration, 135 μm), bimolecular rate constants (*k*_ox_) could be obtained.

### Anaerobic photoreduction and reoxidation

Photoreduction of FsqB was done according to the procedure reported by Massey and Hemmerich (36). Approximately 40 μm of FsqB in 100 mm phosphate/NaOH, pH 7.6, 1 mm EDTA, 1 μm 5-deaza-FMN, and 2 μm methyl viologen were rendered anaerobic by 2-h incubation in an anoxic, nitrogen-filled glove box (Belle Technology). The anoxic samples were transferred to quartz cuvettes and sealed. Photoirradiation was carried out with a 10 W LED flood light (Luminea, Buggingen, Germany), while cooling the cuvette to 15 °C. Spectra were recorded between 300 and 800 nm until no further changes were observed. For reoxidation of the enzyme, the cuvettes were opened to expose the sample to air, and again absorption spectra were recorded between 300 and 800 nm until no further changes were observed.

## Author contributions

M. L., W. K., and P. M. conceptualization; M. L., T. P.-K., M. F., and K. G. data curation; M. L., T. P.-K., M. F., J. N., G. C., B. D., W. K., K. G., and P. M. formal analysis; M. L., T. P.-K., and K. G. validation; M. L., T. P.-K., M. F., J. N., G. C., B. D., and W. K. investigation; M. L., M. F., and J. N. methodology; M. L., W. K., K. G., and P. M. writing-original draft; M. L., W. K., K. G., and P. M. writing-review and editing; T. P.-K., W. K., and K. G. visualization; J. N. software; B. D., W. K., and P. M. supervision; W. K. and P. M. resources; K. G. and P. M. funding acquisition; P. M. project administration.

## Supplementary Material

Supporting Information

## Footnotes

2 The abbreviations used are:

THCS: tetrahydrocannabinol synthase
BBE: berberine bridge enzyme
MTOX: *N*-methyl-tryptophan oxidase
MSOX: (monomeric) sarcosine oxidase
RMSD: root-mean-square deviation
PDB: Protein Data Bank.
