# Supplementary material for: Oxidative cyclization of N-methyl-dopa by a fungal flavoenzyme of the amine oxidase family
Source: J Biol Chem. 2018 Sep 7;293(44):17021–32. doi: 10.1074/jbc.RA118.004227 (PMC6222107; doi:10.1074/jbc.RA118.004227)
Supplement: Supporting Information [file supp_293_44_17021__index.html]

Oxidative cyclization of N-methyl-dopa by a fungal flavoenzyme of the amine oxidase family — Oxidative cyclization of N-methyl-dopa — Oxidative cyclization of N-methyl-dopa by a fungal flavoenzyme of the amine oxidase family — Oxidative cyclization of N-methyl-dopa — Supporting Information 

# Oxidative cyclization of *N*-methyl-dopa by a fungal flavoenzyme of the amine oxidase family

## Supporting Information

- Supporting Information (to be published online) - revised supporting information
