# Supplementary material for: Oxidative cyclization of N-methyl-dopa by a fungal flavoenzyme of the amine oxidase family
Source: J Biol Chem. 2018 Sep 7;293(44):17021–32. doi: 10.1074/jbc.RA118.004227 (PMC6222107; doi:10.1074/jbc.RA118.004227)
Supplement: Supporting Information [file supp_RA118.004227_138492_2_supp_196981_p4kk4n.docx]

**Supporting information**

Oxidative cyclization of *N*-methyl-dopa by a flavoenzyme of the amine oxidase family

**Majd Lahham^$^, Tea Pavkov-Keller^§^, Michael Fuchs^#^, Johannes Niederhauser^$^, Gabriel Chalhoub^$^, Bastian Daniel ^$^, Wolfgang Kroutil^#^, Karl Gruber^§^ and Peter Macheroux^$*^**

^$^Institute of Biochemistry, Graz University of Technology, Petersgasse 12/II, 8010 Graz, Austria

^§^Institute of Molecular Biosciences, University of Graz, Humboldtstr. 50, 8010 Graz, Austria

^#^Institute of Chemistry, University of Graz, Heinrichstr. 28/2, 8010 Graz, Austria

Running title: *Oxidative cyclisation of N-methyl-dopa*

*To whom correspondence should be addressed: Prof. Dr. Peter Macheroux, Graz University of Technology, Institute of Biochemistry, Petersgasse 12/II, A-8010 Graz, Telephone: +43-(0)316-873 6450; FAX: +43 (0)316-873 6952; E-mail: [peter.macheroux@tugraz.at](mailto:peter.macheroux@tugraz.at)

**Table of Contents**

Title page S-1

Synthesis of substrate analogs S-2 – S-4

Table S1: list of primers used for site directed mutagenesis S-5

**Synthesis of substrate analogs**

**General.** All chemicals were from Sigma-Aldrich, Acros Organics or Alfa Aesar and used as received, solvents were obtained from Roth. NMR-spectra were recorded with a Bruker NMR unit at 300 (^1^H) and 75 (^13^C) MHz, shifts are given in ppm and coupling constants (*J*) are given in Hz. High resolution mass spectra were recorded with a Agilent 6230 TOF LC/MS (APCI, positive mode). Optical rotation values were measured with a Perkin Elmer Polarimeter 341. Flash chromatography was performed using Merck silica gel 60 (mesh size 40-63 µm). Petroleum ether had a boiling range of 60-95 °C. The term FsqB-WT refers to the FsqB wild type enzyme. The term FsqB-Var9 refers to variant Asp444Ala.

**Preparation of (*S*)-3-(3,4-dihydroxyphenyl)-2-(methylamino)propanoic acid (4):**

**(*S*)-Benzyl 2-(benzylamino)-3-(3,4-bis(benzyloxy)phenyl)propanoate (2).** L-DOPA (**1**, 1.3 g, 6.6 mmol), potassium carbonate (3.0 g, 21.7 mmol), tetrabutylammonium iodide (420 mg, 1.14 mmol) and sodium iodide (100 mg, 0.67) were suspended in acetone (40 mL) and benzylbromide (3.0 ml, 4.3 g, 25.3 mmol) was added. The mixture was refluxed for 16 h, cooled to room temperature and filtered. The filter cake was washed with additional acetone (40 ml) and the combined filtrate was concentrated. The obtained crude product was purified via flash chromatography (SiO_2_, hexanes/EtOAc 3/1) to give **2** as yellow oil (952 mg, 1.71 mmol, 26%).

[α]_D_^20^ = -6.4 (c 1.2, CHCl_3_); ^1^H-NMR (300 MHz, CDCl_3_): 7.50-7.19 (m, 20H), 6.84 (d, *J* = 8.3, 1H), 6.80 (d, *J* = 2.0, 1H), 6.67 (dd, *J_1_* = 8.3, *J_2_* = 2.0, 1H),5.16 (s, 2H), 5.09 (s, 2H), 5.07 (s, 2H), 3.81 (d, *J* = 13.2, 1H), 3.64 (d, *J* = 13.2, 1H), 3.55 (t, *J* =7.0, 1H), 2.91 (d, *J* = 7.0, 2H); ^13^C-NMR (75 MHz, CDCl_3_): 174.5, 148.8, 147.8, 139.5, 137.4, 137.3, 135.7, 128.6, 128.5, 128.4, 128.32, 128.29, 128.2, 127.8, 127.33, 127.29, 122.2, 116.1, 115.1, 71.4, 71.2, 66.4, 62.0, 52.0, 39.2; IR (film) ῦ = 1730, 1588, 1510, 1454, 1262, 1214, 1162, 1135, 1023, 906, 725, 694; HRMS(APCI): *m*/*z*: calc. for C_37_H_36_NO_4_^+^: 558.2639 [M+H]^+^, found: 558.2634.

**(*S*)-Benzyl 2-(benzyl(methyl)amino)-3-(3,4-bis(benzyloxy)phenyl)propanoate (3).** Compound **2** (883 mg, 1.58 mmol) was dissolved in dichloromethane (22 ml). Anhydrous sodium sulfate (898 mg, 6.32 mmol), sodium triacetoxyborohydride (2.35 g, 11.1 mmol) and formaldehyde (37 wt-% solution in water, 552 µL, 11.7 mmol) were added. The mixture was stirred for 16 h at room temperature. The reaction was quenched by the addition of saturated aqueous ammonium chloride solution (10 ml), the phases were separated, the aqueous phase was washed with EtOAc (2 x 15 ml) and the combined organic phase was dried over sodium sulfate, filtered and concentrated. The crude product was purified via flash chromatography (SiO_2_, hexanes/EtOAc 3/1) to give the target compound **3** as pale yellow oil (881 mg, 1.54 mmol, 98%).

[α]_D_^20^ = -25.3 (c 0.66, CHCl_3_); ^1^H-NMR (300 MHz, CDCl_3_): 7.44-7.10 (m, 20H), 6.82 (d, *J* = 8.2, 1H), 6.76 (d, *J* = 2.0, 1H), 6.66 (dd, *J_1_* = 8.2, *J_2_* = 2.0, 1H), 5.13 (s, 2H), 5.10 (s, 1H), 5.08 (s, 1H), 5.04 (s, 2H), 3.76 (d, *J* =13.9, 1H), 3.58-3.53 (m, 2H), 3.07 (dd, *J_1_* = 13.9, *J_2_* = 7.8, 1H), 2.27 (s, 3H); ^13^C-NMR (75 MHz, CDCl_3_): 171.8, 148.9, 147.6, 139.3, 137.7, 137.5, 136.0, 131.9, 128.8, 128.63, 128.58, 128.55, 128.4, 128.33, 128.27, 127.84, 127.82, 127.4, 127.0, 122.3, 116.3, 115.2, 71.6, 71.3, 67.4, 66.1, 58.8, 38.1, 35.6; IR (film) ῦ = 1727, 1510, 1454, 1262, 1214, 1155, 1167, 1023, 906, 725, 694; HRMS(APCI): *m*/*z*: calc. for C_38_H_38_NO_4_^+^: 572.2795 [M+H]^+^, found: 572.2790.

**(*S*)-3-(3,4-dihydroxyphenyl)-2-(methylamino)propanoic acid (4).** Compound **3** (398 mg, 0.69 mmol) was dissolved in MeOH (10 ml) and Pd/C (10 wt-%, 40 mg) was added. The reaction mixture was stirred vigorously under a hydrogen atmosphere (1 atm.) for 24 h. After removal of the hydrogen atmosphere, HCl (2 vol-% in H_2_O, 10 ml) was added and the mixture was centrifuged and the supernatant was filtered through a syringe filter (PVDF, 33 mm diameter, 0.45 µm). The filter was washed with additional HCl (2 vol-% in H_2_O, 2 x 10 ml). The obtained filtrate was immediately frozen in liquid nitrogen and lyophilized again. Compound **4** was obtained as a colorless solid (112 mg, 0.53 mmol, 77%).

[α]_D_^20^ = -18.2 [c 1.0, HCl (2 vol-% in H_2_O)]; ^1^H-NMR (300 MHz, CDCl_3_): 6.56 (d, *J* = 8.1, 1H), 6.48 (d, *J* = 2.1, 1H), 6.40 (dd, *J_1_* = 8.1, *J_2_* = 2.1, 1H), 3.76 (t, *J* =6.0, 1H), 2.88 (dd, *J_1_* = 6.0, *J_2_* = 5.6, 2H), 2.42 (s, 3H); ^13^C-NMR (75 MHz, CDCl_3_): 170.2, 144.0, 143.4, 125.4, 121.6, 116.6, 116.3, 61.5, 33.8, 31.6; IR (film) ῦ = 3426 (br), 3132 (br); 1570, 1481, 1386, 1294, 1200, 780, 542, 527; HRMS(APCI): *m*/*z*: calc. for C_10_H_14_NO_4_^+^: 212.0917 [M+H]^+^, found: 212.0917.

**Preparation of *rac*-3-(3-hydroxyphenyl)-2-(methylamino)propanoic acid (9):**

**(*rac*)-2-{[(benzyloxy)carbonyl]amino}-3-(3-{[(benzyloxy)carbonyl]oxy}phenyl)propanoic acid (6).** *meta*-Tyrosine (**5**, 1.0 g, 5.5 mmol) was dissolved in NaOH_aq._ solution (11 ml, 0.5 M) and cooled to 4°C. A solution of benzyl chloroformate (1.88 g, 11 mmol, 1.57 ml, 2 equiv.) in Et_2_O (15 ml) and a NaOH_aq._ solution (19 ml, 1.00 M) were added dropwise simultaneously over a period of 1 h. The reaction mixture was stirred for 1 h at 4 °C and 2 h at room temperature. The obtained slurry was filtered and the pH of the filtrate was adjusted to 1-2 by the addition of solid citric acid. The aqueous phase was extracted with Et_2_O (3 x 50 ml), the combined organic phase was dried over Na_2_SO_4_, filtered and concentrated to give a yellow oil (2.3 g), which was directly used for the next reaction step without further purification.

**(*rac*)-methyl 2-{[(benzyloxy)carbonyl](methyl)amino}-3-(3-{[(benzyloxy)carbonyl]oxy}phenyl)propanoate (7).** Crude product **6** (2.3 g, 5.1 mmol) was dissolved DMF (10 ml), Cs_2_CO_3_ (5.4 g, 16.5 mmol) and iodomethane (4.68 g, 33 mmol, 2.05 ml) were added. The reaction mixture was stirred for 16 h at 90 °C. The reaction mixture was cooled to room temperature and quenched by the addition of NH_4_Cl_aq., sat._ solution (10 ml). The mixture was extracted with EtOAc (3 x 50 ml), the combined organic phase was dried over Na_2_SO_4_, filtered and concentrated to give a crude product oil, which was purified via flash chromatography (SiO_2_, hexanes/EtOAc 3/1) to give **7** as a colorless oil (245 mg, 0.51 mmol, 10%), which was inseparable from EtOAc (ca. 25% according to NMR). The low yield is due to partial de-protection of the carbonate group and methylation of the deliberated phenol under the reaction conditions.

^1^H-NMR (300 MHz, CDCl_3_): 7.43-7.21 (m, 11H), 7.09-6.99 (m, 3H), 5.25 (s, 2H), 5.09 (s, 1.2H, rotamer 1), 5.04 (s, 0.8H, rotamer 2), 4.91 (dd, *J_1_* = 10.6, *J_2_* = 5.4, 0.6H, rotamer 1), 4.73 (dd, *J_1_* = 10.4, *J_2_* = 4.9, 0.4H, rotamer 2), 3.73 (s, 1.8H, rotamer 1), 3.65 (s, 1.2H, rotamer 2), 3.39-3.27 (m, 1H), 3.12-2.94 (m, 1H), 2.82 (s, 1.2H, rotamer 2), 2.79 (s, 1.8H, rotamer 1); ^13^C-NMR (75 MHz, CDCl_3_): 171.3 (rotamer 1), 171.1 (rotamer 2), 156.6 (rotamer 1), 155.9 (rotamer 2), 153.7 (rotamer 1), 153.6 (rotamer 2), 151.33 (rotamer 2), 151.26 (rotamer 1), 139.2 (rotamer 2), 139.1 (rotamer 1), 136.7, 136.4, 134.9, 129.7, 128.9 (rotamer 2), 128.8 (rotamer 1), 128.7 (rotamer 1), 128.6 (rotamer 2), 128.2 (rotamer 2), 128.1 (rotamer 1), 128.0, 127.7, 126.70 (rotamer 2), 126.67 (rotamer 1), 121.7 (rotamer 1), 121.6 (rotamer 2), 119.6 (rotamer 2), 119.5 (rotamer 1), 70.4, 67.6 (rotamer 2), 67.4 (rotamer 1), 60.8 (rotamer 2), 60.4 (rotamer 1), 52.5, 35.2 (rotamer 1), 34.8 (rotamer 2), 32.6 (rotamer 1), 32.2 (rotamer 2); IR (film) ῦ = 3065, 3033, 2952, 1759, 1742, 1698, 1487, 1452, 1400, 1379, 1314, 1218, 1137, 1002, 838, 768, 735, 695; HRMS(ESI): *m*/*z*: calc. for C_27_H_28_NO_7_^+^: 478.1860 [M+H]^+^, found: 478.1857.

**(*rac*)-methyl 2-{[(benzyloxy)carbonyl](methyl)amino}-3-(3-{[(benzyloxy)carbonyl]oxy}phenyl)propanoate (6). 7** (225 mg, 0.47 mmol) was dissolved THF (5 ml) and NaOH_aq._ solution (1.8 ml, 3.5 M) was added. The reaction mixture was stirred for 32 h at room temperature. The pH of the mixture was adjusted to 1 with HCl (6M) and the slurry was extracted with Et_2_O (3 x 20 ml). The combined organic phase was dried over Na_2_SO_4_, filtered and concentrated. The obtained crude product was directly used for the next reaction step, without further purification.

**(*rac*)-3-(3-hydroxyphenyl)-2-(methylamino)propanoic acid (9).** Crude product **8** (153 mg, 0.47 mmol) was dissolved in MeOH (5 ml). Pd/C (10 wt-% Pd, 20 mg) was added and the reaction mixture was vigorously stirred under a hydrogen atmosphere (1 atm.). After 6 h the hydrogen atmosphere was removed and hydrochloric acid (2M, 2 ml) was added. The obtained slurry was filtered through a syringe filter (PVDF, 33 mm diameter, 0.45 µm). The filter was washed with additional hydrochloric acid (2M, 2 x 4 ml) and the combined filtrate was frozen in liquid nitrogen and lyophilized. Compound **9** was obtained as a pale yellow solid (72 mg, 0.37 mmol, 79% over 2 steps).

^1^H-NMR (300 MHz, D_2_O + DCl): 7.26 (t, *J* = 7.9, 1H), 6.85 (d, *J* = 2.1, 1H), 6.82 (d, *J* = 2.0, 1H), 6.76 (t, *J* = 1.9, 1H), 4.17 (t, *J* = 4.2, 1H), 3.73 (dd, *J_1_* = 6.1, *J_2_* = 4.3, 1H), 2.71 (s, 3H); ^13^C-NMR (75 MHz, D_2_O + DCl): 170.9, 155.9, 135.4, 130.5, 121.4, 116.1, 114.9, 62.3, 34.8, 31.9; IR (film) ῦ = 3528 (br), 3413 (br), 3285 (br), 2612, 2426, 2207, 2128, 1729, 1595, 1492, 1451, 1404, 1278, 1245, 1229, 1164, 1073, 877, 794, 699, 463, 440; HRMS(ESI): *m*/*z*: calc. for C_10_H_14_NO_3_^+^: 196.0968 [M+H]^+^, found: 196.0970.

**Table**

**Table S1: list of primers used for site directed mutagenesis.**

| **Amino acid replacement** | **Forward primer** | **Reverse primer** |
| --- | --- | --- |
| R63M | CAGATACCAGCATGGTTATTCGTCGTGATTATC | GACGAATAACCATGCTGGTATCTGCATTTG |
| R66M | CCAGCCGTGTTATTATGCGTGATTATCCGCATG | ATAACACGGCTGGTATCTGCATTTGCTGCTG |
| Y121F | CCGTGAATTTCATCAAAAAAGCCTATGCCATTAGCTGCGAACTG | GCTTTTTTGATGAAATTCACGGTTTCCAGTGCTTTCGGAGG |
| K304A | GTCTGGCTTTTGGCCACTTTAGCTATAGCGGTATTGTTGATGTTCTG | GTGGCCAAAAGCCAGACAATTATCATGATCCGGACCAACTG |
| Y416F | GTTGGTTCAACGATACACCGGCACTGGATTTTGTTGTTGATTATCATC | CGGTGTATCGTTGAACCAACATTTACGAACACGGGTAAACGG |
| D444A | GCGACCGGTGGTTGTGCACATGCATTCAAATTTCTG | CACCGGTCGCAACAAACAGGGTTTTGCCATAGC |
| K448A | GCATTCGCTTTTCTGCCGATTATTGGTGAAAAAACCCTGGC | GGCAGAAAAGCGAATGCATGATCACAACCACCGGTC |
